# Outcome analysis of brain-death referral to NOD-Lb: A retrospective chart review of a single hospital experience over 3 years

**DOI:** 10.1371/journal.pone.0295930

**Published:** 2024-02-13

**Authors:** Hachem Araji, Johnny Ayoub, Laudy Gebrael, Hiba Fala, Elio Junior Feghali, Marwa Al Jardali, Sleiman Iskandar, Yana Said, Maria Nakhoul, Wissam Faour, Sola Aoun Bahous, Farida Younan, Antoine Stephan

**Affiliations:** 1 Gilbert & Rose-Marie Chagoury School of Medicine, Lebanese American University, Byblos, Lebanon; 2 Dana-Farber Cancer Institute, Department of Informatics and Analytics, Boston, Massachusetts, United States of America; 3 National Organization for Organ and Tissue Donation and Transplantation (NOD-Lb), Baabda, Lebanon; University Medical Centre Ljubljana (UMCL) / Faculty of Medicine, University Ljubljana (FM,UL), SLOVENIA

## Abstract

**Background:**

Organ donation shortage and in particular organ procurement is an international concern as the gap between the number of donors and recipients is steadily growing. Organ procurement is a chain of steps with donor identification and referral (ID&R) as the very first link in this chain. Failure of this step hinders the progress in the organ transplantation program.

**Objectives:**

Our study was conducted to evaluate and highlight the gap between the national system and the practice at the identification and referral (ID&R) step of the organ procurement chain in a single tertiary-care academic health center in Beirut: the Lebanese American University Medical Center–Rizk Hospital (LAUMC-RH), and to appraise the literature for challenges at this step and for possible interventions for improvement based on the international experience.

**Materials and methods:**

This retrospective study was a descriptive case series of ICU and ED deceased patients at a single tertiary-care university hospital in Beirut. Patients’ characteristics were collected from medical records for all patients who died between 2017 and 2019 while in the ICU or the ED and shared with the National Organization for Organ and Tissue Donation and Transplantation (NOD-Lb), for each subject separately, to decide on the donor status. All data collected from the patient cohort was analyzed using R version 3.6.1. Wilcoxon signed-rank test, chi-squared, and fisher-exact tests were used to compare differences in clinical characteristics in terms of donor status when appropriate.

**Results:**

This study served as 3 years audit of a single hospital experience, and it demonstrates failure to make any referrals to NOD-Lb and zero actual organ and tissue donations over the study period. The review of 295 deceased subjects’ charts demonstrates 295 missed alerts to NOD-Lb and the overall missing of 5 organ and tissue donors and 24 cornea donors assuming the organ procurement chain of steps will continue uninterrupted after ID&R.

**Conclusion:**

The data gathered suggests the presence of an inefficient identification and referral system that is translated into a complete failure of reporting to NOD-Lb from LAUMC-RH. A systematic evidence-based approach to evaluate for the most cost-effective intervention to increase identification and referral rates is needed with a serious effort to examine and account for any inefficient implantation.

## Introduction

Organ donation shortage and in particular organ procurement is an international concern as the gap between the number of donors and recipients is steadily growing. Around 105,800 people were added to the U.S. national transplant waiting list by December 2022, whereas only about 40,000 transplantations were actually performed in 2021 [1]. It is important to note that there are two main types of organ donations: Living and Deceased donations.

Deceased organ donation is of two types: donation after death by neurologic criteria (DNC) and donation after cardiac death (DCD) [2]. DCD was the standard practice before the concept of DNC (or brain death) was established [3]. A prerequisite to DNC is the confirmation of brain death diagnosis [4]. DNC rate is dependent on: public acceptance of the concept of brain death, healthcare providers proficiency in diagnosing DNC, healthcare workers attitudes and knowledge of the organ donation process, and communication with the family of the deceased [5].

It is worth noting that not all patients are properly identified as brain dead, and in the case they were, their referral to the Organ Procurement Network is expected but not always applied [6].

Lebanon is an example where the number of patients on the waiting list for transplantation largely exceeds the number of eligible donors. The latest data from 2018 showed that 4 kidneys, 1 liver, 2 hearts, and 1 lung from deceased donors were successfully transplanted whereas 101 kidneys and 8 livers from living donors were performed in the same year [7].

In Lebanon deceased organ donation is restricted to DNC; only tissues (corneas) can be obtained several hours after cardiopulmonary arrest. Organ donation in Lebanon relies on the national system established by a non-governmental organization known as the National Organization for Organ and Tissue Donation and Transplantation (NOD-Lb). This organization is affiliated to the ministry of health and has the role of setting organ donation protocol guidelines, coordinating the organ procurement process, managing waiting lists, educating healthcare professionals, raising public awareness, and monitoring performance at the national level [8]. An opt-in system where donors must actively sign to a register to donate their organs after death is adopted in Lebanon, with the national donor registry operated by NOD-Lb. It is worth noting that signing a donor card in Lebanon is not legally binding unless family consent is also granted after the death of the donor [8].

The National Organ Procurement Network is established and supervised by NOD-Lb as the central office collaborating with 23 affiliated hospitals from different regions in Lebanon. Today, Lebanon has 15 solid organ transplantation units. Currently, LAUMC-RH is part of the national organ procurement network, but it does not run a transplantation unit [8].

For deceased organ donation, data from NOD-Lb, for the years 2017 to 2019 demonstrates 185 referrals with 105 eligible donors compared to 376 patients registered on the national waiting list for kidney, liver and heart transplantation [8]. Table 1 details the critical pathway for deceased organ donors in Lebanon from 2017 to 2019 [8].

**Table 1:** Critical pathway for deceased organ donors, Lebanon 2017–2019 (NOD-Lb).

Population	2017	2018	2019	Total
	5 million	5 million	5 million	
**Potential Donor**	53	53	55	161
**Referrals from LAUMC-RH**	0	0	0	0
**Eligible Donor**	41	28	36	**105**
**Families approached**	30	19	16	65
**Families consented**	13	4	6	23
**Actual Donor**	13	4	6	**23**
**Utilized Donor**	8	2	2	**12**
**Conversion Rate**	3	0.9	1.4	

*Note*. **Potential Donor** (Person whose clinical condition is suspected to fulfil brain or circulatory death criteria), **Eligible Donor** (Medically suitable person who has been declared brain dead), **Actual Donor** (Deceased person from whom at least one organ has been recovered), **Utilized Donor** (Actual donor from whom at least one organ was transplanted), **Conversion Rate** (Number of actual donors divided by the number of potential donors), Lebanese American University Medical Center–Rizk Hospital (LAUMC-RH).

Organ procurement is a chain of steps starting with identification and referral (ID&R). According to NOD-Lb, in case of potential organ donation following neurologic criteria, the steps are the following: Identification and referral → Brain death diagnosis → Donor maintenance → Organ viability → Family consent → Organ allocation → Organ retrieval → Organ transplantation. The very first link in this chain is detection and referral. The Deceased Alert System (DAS) was introduced by NOD-Lb to account for the timely identification and referral of potential organ donors [8]. Failure of this step hinders the progress in the organ transplantation program [4].

Our study was conducted to evaluate and highlight the gap between the national system and the practice at the identification and referral (ID&R) step of the organ procurement chain in a single tertiary care academic health center in Beirut, the Lebanese American University Medical Center–Rizk Hospital (LAUMC-RH), and to appraise the literature for challenges at this step to inform corrective actions.

## Methods

### Study design and setting

This is a retrospective study involving patients who died in the Intensive care Unit (ICU) or the Emergency Department (ED) between 2017 and 2019 at LAUMC-RH, a tertiary care academic health center in Beirut, Lebanon. Patients’ characteristics were collected from medical records for all patients and shared with the National Organization for Organ and Tissue Donation and Transplantation (NOD-Lb).

The deceased alert system (DAS) is a national monitoring and reporting program for cardiac death and death by neurological criteria set in place to detect potential deceased donors at all Lebanese hospitals. It is a system established by NOD-Lb and adopted by Lebanese Ministry of Health and is used to detect 100% of the deaths in a Lebanese hospital (in the ICU and ED) regardless of whether the deceased would be eligible for organ donation after accounting for medical contraindications.

The criteria of DAS are:

For possible donors following death by neurological criteria:

- Severe neurological damage
- Glasgow Coma Scale ≤ 5
- On mechanical ventilation

For possible tissue donors after cardiopulmonary arrest:

- any patient with cardiorespiratory arrest.

### Sample

Our sample included all 295 deceased patients for the three years period from 2017 to 2019.

### Variables

The list of criteria to screen patients’ files for eligibility as potential organ donor was provided by NOD-lb. The list included documentation of absolute contraindications (HIV status, HBV status, Uncontrolled sepsis, Multi-organ failure, Cancer, Prion disease, Alzheimer’s disease, HCV status), documentation of relative contraindications (Age, Diabetes, Hypertension, and Uncontrolled infections), documentation of clinical criteria that warrant identification and referral (DAS system criteria: severe neurological damage, Glasgow Coma Scale ≤5, on mechanical ventilation, and any patient with cardiopulmonary arrest), and documentation of the underlying cause of death. Other criteria screened for included documentation of the level of care (ICU or ED), documentation of code status (Do not intubate (DNI), Do not intubate and do not resuscitate (DNI/DNR), palliative care), and finally documentation of brain death (BD) diagnosis.

Data collected were shared with NOD-Lb, for each subject separately, to decide on the donor status as per the following: non-donor, organ and tissue donor, cornea donor, or probable organ and tissue donor because of missing data.

**Fig 1:**
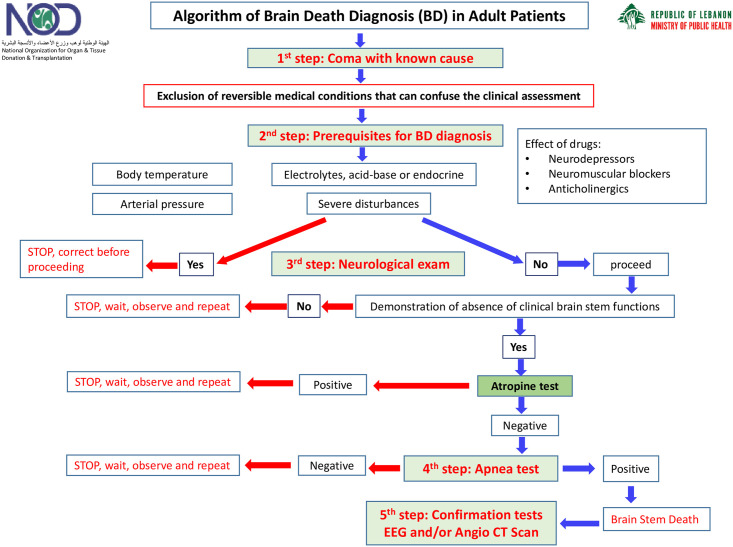
Algorithm of brain death diagnosis in adult patients.

For the sake of this retrospective study: Non-donors are subjects with at least one absolute contraindication that precludes organ/tissue donation. Cornea donors are patients with cardiopulmonary arrest and with no documented contraindication for cornea donation. Organ and tissue donors are subjects with BD diagnosis or suspected to fulfill BD diagnosis criteria according to the national guidelines (Fig 1) and with no documented contraindication for organ and tissue donation.

### Statistical analysis

All data collected from the patient cohort was analyzed using R version 3.6.1. Patient characteristics were described with categorical variables represented as n (%) and continuous variables were represented as median with interquartile range (IQR). Wilcoxon signed-rank test, chi-squared, and fisher-exact tests were used to compare differences in clinical characteristics in terms of donor status when appropriate. A p-value of less than or equal to 0.05 was considered statistically significant.

### Ethical considerations

Patients’ records were treated with confidentiality. No personal information was disclosed. Anonymity was preserved in a way that no names were revealed throughout the study and any information that would possibly unravel the patient’s identity was removed. This study was approved by the LAU-IRB.

## Results

A total of 295 patients were included in the analysis.

Table 1 demonstrates the evolvement of the deceased donor process in Lebanon between 2017 and 2019 nationally and the contribution of the academic tertiary care health center in Beirut LAUMC-RH [8]. Zero referrals were issued from LAUMC-RH during the study period.

Table 2 highlights the differences in the proportions of demographic and clinical characteristics among donors and non-donors. The mean age of patients at the time of the study was 62 years for donors (SD±22.77) and 77 years for non-donors (SD±0.97).

**Table 2:** Characteristics of donors and non-donors.

Variable	Donor (n = 31)	Non-Donor (n = 264)	*p*-value
**Age, years**	62 (22.77)	77 (0.97)	**<0.0001**
**Gender**			0.3
Female	10 (32.3%)	116 (43.9%)	
Male	21 (67.7%)	148 (56.1%)	
**HIV status**			NA
Not documented	8 (25.8%)	39 (14.8%)	
Negative	23 (74.2%)	225 (85.2%)	
Positive	0 (0%)	0 (0%)	
**HBV**			1
Not documented	8 (25.8%)	39 (14.7%)	
Negative	23 (74.2%)	223 (84.5%)	
Positive	0 (0%)	0 (0%)	
**Uncontrolled sepsis**			**<0.0001**
Not documented	8 (25.8%)	38 (14.4%)	
Negative	23 (74.2%)	128 (48.5%)	
Positive	0 (0%)	98 (37.1%)	
**Multi-organ failure**			**0.0001**
Not documented	8 (25.8%)	38 (14.4%)	
Negative	20 (64.5%)	103 (39.0%)	
Positive	3 (9.7%)	123 (46.6%)	
**Cancer**			**0.0004**
Not documented	8 (25.8%)	33 (12.6%)	
Negative	22 (71%)	130 (49.2%)	
Positive	1 (3.2%)	97 (36.7%)	
History of cancer	0 (0%)	4 (1.5%)	
**Prion disease**			NA
Not documented	8 (25.8%)	38 (14.4%)	
Negative	23 (74.2%)	226 (85.6%)	
Positive	0 (0%)	0 (0%)	
**Alzheimer’s disease**			1
Not documented	8 (25.8%)	37 (14%)	
Negative	23 (74.2%)	219 (83%)	
Positive	0 (0%)	8 (3%)	
**HCV**			1
Not documented	8 (25.8%)	39 (14.7%)	
Negative	23 (74.2%)	223 (84.5%)	
Positive	0 (0%)	2 (0.8%)	
**Diabetes**			1
Not documented	8 (25.8%)	37 (14%)	
Negative	14 (45.2%)	137 (51.9%)	
Positive	9 (29%)	90 (34.1%)	
**Hypertension**			**0.022**
Not documented	8 (25.8%)	35 (13.3%)	
Negative	13 (41.9%)	70 (26.5%)	
Positive	10 (32.3%)	159 (60.2%)	
**Uncontrolled infections**			**<0.0001**
Not documented	8 (25.8%)	40 (15.2%)	
Negative	21 (67.7%)	111 (42%)	
Positive	2 (6.5%)	113 (42.8%)	
**Level of care**			0.1
ER	13 (41.9%)	72 (27.3%)	
ICU	18 (58.1%)	192 (72.7%)	
**DNR & DNI positive**			0.1
Not documented	0 (0%)	0 (0%)	
No	28 (90.3%)	186 (70.5%)	
Yes	1 (3.2%)	28 (10.6%)	
Palliative care term used	2 (6.5%)	49 (18.6%)	
DNI only	0 (0%)	1 (0.4%)	
**BD Diagnosis documented**			0.1
No	30 (96.8%)	264 (100%)	
Yes	1 (3.2%)	0 (0%)	

*Note*. HIV (*Human Immunodeficiency Virus)*, HBV (*Hepatitis B Virus)*, HCV (*Hepatitis C Virus)*, Do Not Resuscitate (DNR), Do Not Intubate (DNR), Brain Death (BD)

Non-donors were significantly more likely to have uncontrolled sepsis (37.1% vs. 0%; p< 0.0001) than donors, as well as multi-organ failure (46.6% vs. 9.7%; p = 0.0001) and cancer (36.7% vs. 3.2%; p = 0.0004) respectively. Also, non-donors were significantly more likely to have hypertension (60.2% vs. 32.3%; p = 0.022), and uncontrolled infections (42.8% vs. 6.5%; p <0.0001) compared to donors respectively.

Table 3 reveals that 89.50% (n = 264) of subjects did not meet the criteria to become organ or tissue donors. However, 8.13% (n = 24) were cornea donors, while 1.69% (n = 5) were organ and tissue donors. There were zero actual donors from LAUMC-RH in the study sample over the study period. The analysis included potential donors.

**Table 3:** Frequency distribution of donor status.

Donor status	N	%
Non-donor	264	89.50%
Cornea Donor	24	8.13%
Organ and Tissue Donor	5	1.69%
Probable Organ and Tissue Donor	2	0.68%

Table 4 shows the distribution of donor status through the years 2017–2019. The year 2018 had the highest number of organ or tissue donors (n = 16), followed by 2017 (n = 12) and lastly, 2019 (n = 3).

**Table 4:** Donor status by year.

Donor Status	2017	2018	2019
Donor	12 (38.7%)	16 (51.6%)	3 (9.7%)
Non-Donor	94 (35.6%)	113 (42.8%)	57 (21.6%)

Table 5 highlights the difference in proportions of meeting DAS referral criteria and in the number of DAS alerts received by NOD-Lb between donors and non-donors. Zero alerts were received by NOD-Lb from LAUMC-RH over the study period.

**Table 5:** DAS criteria and alerts received by NOD-Lb for donors and non-donors.

Variables	Donor (n = 31)	Non-Donor (n = 264)	*p*-value
**1. Severe neurologic damage**			0.06
Not documented	10 (32.3%)	45 (17%)	
No	11 (35.5%)	162 (61.4%)	
Yes	10 (32.3%)	57 (21.6%)	
**2. Glasgow comma scale ≤ 5**			0.3
Not documented	12 (38.7%)	63 (23.8%)	
No	12 (38.7%)	152 (57.6%)	
Yes	7 (22.6%)	49 (18.6%)	
**3. On mechanical ventilation**			1
Not documented	8 (25.8%)	25 (9.5%)	
No	9 (29%)	92 (34.8%)	
Yes	14 (45.2%)	147 (55.7%)	
**1, 2, & 3 positive**			0.2
Not documented	9 (29%)	25 (9.6%)	
No	15 (48.4%)	195 (73.7%)	
Yes	7 (22.6%)	44 (16.7%)	
**Cardiopulmonary Arrest**			0.8
Not documented	0 (0%)	0 (0%)	
No	0 (0%)	3 (1.1%)	
Yes	29 (93.5%)	235 (89%)	
Arrived dead	2 (6.5%)	26 (9.9%)	
**DAS alert received**			N/A
No	31 (100%)	264 (100%)	
Yes	0 (0%)	0 (0%)	

*Note*. Disease Activity Score (DAS)

## Discussion

This study served as 3 years audit of a single hospital experience, and it demonstrates failure to make any referrals to NOD-Lb and zero actual organ and tissue donations.

According to the deceased organ procurement system in Lebanon, each hospital should have a hospital organ procurement unit [9]. Such a unit is responsible to refer **every** critical patient fulfilling the 3 criteria of a possible brain death donor and every cardiopulmonary arrest patient admitted to the ICU and ED only. NOD-Lb will evaluate each referred subject for relative and absolute contraindications to decide on the suitability of a potential donor. NOD-Lb becomes on board from the referral to the end of the procedure.

The DAS system was introduced by NOD-Lb to account for the timely identification and referral of potential organ donors as part of a solid organ procurement infrastructure [10] and organ procurement protocols were added to the national criteria for accreditation of Lebanese hospitals [11].

The review of 295 deceased subjects’ charts demonstrates 295 missed alerts to NOD-Lb and the overall missing of 5 organ and tissue donors and 24 cornea donors. Among the 5 organ and tissue donors (subjects with BD diagnosis or suspected to fulfill BD diagnosis criteria according to the national guidelines and with no documented contraindication for organ and tissue donation) BD diagnosis was declared in only 1 patient. All 5 organ donor patients (Organ donors as subjects with BD diagnosis or suspected to fulfill BD diagnosis criteria and with no documented contraindication for organ and tissue donation) were eventually declared circulatory death highlighting a failure in organ and tissue procurement from brain-dead/potentially BD donors before cardiac arrest ensues.

Table 4 shows considerable variation in potential donors between different years. More than half were reported in 2018. No specific reason can historically explain this variation based on the context review.

In Lebanon deceased organ donation is restricted to DNC (donation after death by neurologic criteria) and Lebanon strictly follows the dead donor rule. It is worth mentioning that absolute and relative contraindications were only documented retrospectively after chart review. Absolute and relative contraindications were not by themselves a reason for not donating as the chain of events ended at the 1st step which is identification and referral.

On the global level, three main challenges face the identifying and referral step: inconsistent definition of a potential donor, lack of favorable referral legislation, and end-of-life care and donation topic-associated discomfort [12].

Multiple criteria are utilized to define and detect potential donors in Lebanon and worldwide with a lack of unified international criteria set. Common elements include mechanical ventilation, low Glasgow Coma Scale, end-of-life discussions, severe neurological damage, and brain death [12].

In Lebanon, there are well-defined criteria for possible and potential deceased organ donors after brain death. A **possible deceased organ donor** is defined as a patient with a devastating brain injury or lesion or a patient with circulatory failure and apparently medically suitable for organ donation. A **potential Donation after Brain Death (DBD) donor** is defined as a person whose clinical condition is suspected to fulfill brain death criteria.

A multiregional working group in collaboration with the WHO, The Transplantation Society (TTS), and the Spanish Organizacion Nacional de Trasplantes (ONT) established the critical pathway, a universal systematic approach to the process of deceased organ donation, which included a universal agreement on the definitions of potential deceased organ donors and on the creation of trigger for identification and referral [13].

In a systematic review published by Squires et al. aimed at appraising the published literature for the definitions of a potential deceased organ donor and the clinical criteria used for identification, no one global definition or a unified set of clinical criteria was identified [2].

In a study based in Poland on the potential of organ donation from various hospitals, a hospital stratification system that takes into account each hospital’s resources (e.g. presence or not of a neurology department…) was created to calculate such potential. It was proposed that hospitals within the same stratum would have the same potential. To test this assumption hospitals within one stratum were evaluated for donation activity. Results showed that among 19 hospitals with similar profiles the donation activity varied from 0 to 62 donations in a 3-year period between different hospitals. Such stratification system was suggested to help in estimating the number of lost potential donors from active and non-active hospitals when analyzing historical data [14].

In the potential donor audit report for the 12-month period (1 April 2021–31 March 2022) that included all audited patient deaths in UK ICUs and ED, the major reason why potential donors weren’t referred is the lack of identification [15]. In the same report, of the patients who met the referral criteria for Eligible donors after brain death (DBD) and/or Eligible donors after circulatory death (DCD), 99% and 90% were referred to NHS Blood and Transplant [15].

In the UK, the NICE 2011 [16] and GMC 2010 guidelines [17] make organ donation consideration the standard practice in end-of-life care. These guidelines are brought into practice by encouraging hospitals to develop clinical pathways for the timely identification and referral of potential organ donors who meet clinical triggers [16].

In UAE, where deceased organ donation process was initiated in 2016, the lack of proper ID&R protocols accounted for a major obstacle to the organ donation process based on a single hospital retrospective chart review study over one year, whereby none of 20 subjects confirmed as eligible organ donors (subjects with brain death and no medical contraindication), was converted to actual donors.

In Canada, ICU physicians take an important part in the identification and referral of potential donors [18]. Missed donor ID&R is an ongoing problem in Canada even though many Canadian provinces have required referral legislation and a set definition of a potential organ donor [12] highlighting a gap at the level of implementation [18]. In a self-administered survey of Canadian intensivists to evaluate attitudes on physician non-referral (physicians electing not to refer potential organ donor subjects): 44% of participants elected not to refer potential donors in the past, 59% of them did so because of the assumption that donation would not proceed because of organ dysfunction, and 42% did so because they considered the family was too stressed to consider organ donation. None of the participants reported holding a personal belief against organ donation [18].

A systematic review published by Witjes et al. on the effect of the different interventions on increasing the number of organ donors found that identification and referral rates improve through a collaborative care approach between different departments aimed at early identification of clinical triggers [19].

One intervention that resulted in a statistically significant increase in identification rates was an intervention that introduced a multidisciplinary approach to institutionalize organ donation protocols in 50 hospitals. The intervention entailed feedback on previous hospital performance in organ donation practices, staff training, and monitoring [20]. A 3 phases intervention over 13 years implemented by Santa Catarina Brazilian state over 13 years resulted in a statistically significant increase in the number of referrals. A multimodal action plan adopted from the Spanish model of transplant coordination was implemented progressively. Among the actions taken were: initiating a quality improvement program, establishing in-house transplant coordination teams, and introducing training programs for healthcare professionals on different aspects of organ donation [21].

Besides ICUs, end-of-life decision-making discussions are also part of the ED clinical encounters. Like the ICU setting, patients in the ED can rapidly decompensate and die. As such, timely reporting of patients meeting clinical triggers for potential organ donors is important. Missed identification and referral of potential organ donors from the ED is attributed to factors related or not to the ED setting [22]. Among the former factors are the crowding [22] and the limited time and resources in an ED setting [23]. Among the latter are factors related to knowledge of and attitudes toward organ donation and the lack of communication between EDs and organ procurement units [22].

In 2016, the Spanish Organizacion Nacional de Trasplantes (ONT) in collaboration with the Spanish Society of Emergency Medicine issued guidelines that allow the timely identification of potential organ donors in the ED [24]. These guidelines specified the responsibilities of emergency professionals in identification and referral including communication with families and transplant coordinators complemented with the necessary training for ED physicians and staff [24]. Intensive training for ED staff on organ procurement protocols [25], and the utilization of embedded specialist nurse with a collaborative care approach in the ED for identification and referral are two interventions that resulted in a statistically significant increase in the referral rate from the ED [26].

Lebanon was one of the three countries in the MENA region where the European-Mediterranean Postgraduate Programme on Organ Donation and Transplantation (EMPODaT) project was implemented. This project resulted in improvement of the healthcare sciences postgraduate students’ knowledge of living and deceased organ donation topics in the three countries. However, the impact of this training on organ donation and transplantation practice couldn’t be demonstrated due to the lack of reliable quality improvement protocols in these countries [27]. Moreover, prior and throughout the study period, NOD-Lb has maintained its annual program of educating, raising awareness and supporting hospitals involved in the organ and tissue donation program. For example, activities reaching out to the public, college and university students, and healthcare professionals were organized and administered on a regular basis [8].

The gap between the national system and the practice at the identification and referral (ID&R) step of the organ procurement chain highlighted in our study is only one challenge in a multifaceted process. In her recently published doctoral thesis, based primarily on a qualitative research design, Stephan J. addresses the process of organ donation and transplantation in Lebanon through the performance management lens. In her performance analysis of the organ donation and transplantation process, she focuses on service delivery, governance, and society. The author identifies several drawbacks at each of the three levels. On the service delivery level, she describes delays in the legal process, poor communication with the parents, inefficient organ maintenance, and incomplete reporting among others. On the governance level, she mentions outdated and loosely formulated laws, inadequate financial support, and deficiencies in materials and infrastructure to mention some. On the societal level, she sheds light on the lack of support from health professionals, religion, lack of information and commitment from the public, and more. From her thesis analysis, she suggests multiple recommendations at the NOD-Lb, Ministry of Public Health (MoPH), and the society levels. One recommendation at the societal level is that health professionals and hospital administrators should invest more and participate more actively in the process. At the ministry level, performance management systems should be developed to include a solid strategic plan with objectives, performance indicators, and performance evaluation mechanisms. At NOD-Lb level, the coordination with MoPH must take a formal structure. NOD-Lb must be empowered by the legal means and resources to implement the process of organ donation as a private entity in the spirit of a public-private partnership [28].

The major limitation of our study is the fact that it is based on a single hospital experience. Therefore, results don’t necessarily reflect the practice at other national healthcare centers. Moreover, as this study is retrospective, some of the medical records were incomplete in terms of the variables of interest. A data entry sheet for the variables of interest with options for each variable was designed and filled for each subject to ensure comparable screening of files. A significant proportion of the absolute and relative contraindications to donation were not documented indicating that the chart documentation isn’t optimized for organ donation auditing purposes. Furthermore, we did not survey the healthcare professionals and administrators involved in decision-making at the study site to understand the major factors challenging identification and referral, which would have helped in setting-up a corrective action.

In conclusion, this study aims to evaluate for missed potential organ donors and helps to highlight the gap in the national protocols’ implementation and the consequence of the lack thereof on a local level. The data gathered suggests the presence of an inefficient identification and referral system that is translated into a complete failure of reporting to NOD-Lb. But also, lack of proper day-to-day communication between NOD-Lb and the concerned hospitals. While in other countries, organ transplant coordinator act as intermediate to identify potential, such system is still lacking in Lebanon. While NOD-Lb is legitimized by law to implement the process of organ donation it has no legal authority to impose rules and regulations especially since the healthcare sector in Lebanon is predominantly a private entity [28]. Therefore, a systematic evidence-based approach to evaluate for the most cost-effective intervention to increase identification and referral rates is needed with a serious effort to examine and account for any inefficient implantation. Lastly, optimized data archives, perhaps with a section on organ donation potential in each medical record, could be established to better utilize this archive in future organ procurement quality improvement projects.

## References

[1] government U. U.S. government information on organ donation and transplantation.

[2] Squires JE, Coughlin M, Dorrance K, Linklater S, Chassé M, Grimshaw JM, et al. Criteria to Identify a Potential Deceased Organ Donor: A Systematic Review. Critical care medicine. 2018;46(8):1318–27. Epub 2018/05/22. doi: 10.1097/ccm.0000000000003200 . 29782354

[3] Maciel CB, Hwang DY, Greer DM. Chapter 23—Organ donation protocols. In: Wijdicks EFM, Kramer AH, editors. Handbook of Clinical Neurology. 140: Elsevier; 2017. p. 409–39. 28187813 10.1016/B978-0-444-63600-3.00023-4

[4] Manara AR, Thomas I. Current status of organ donation after brain death in the UK. Anaesthesia. 2020;75(9):1205–14. Epub 2020/05/21. doi: 10.1111/anae.15038 . 32430995

[5] Kosieradzki M, Jakubowska-Winecka A, Feliksiak M, Kawalec I, Zawilinska E, Danielewicz R, et al. Attitude of healthcare professionals: a major limiting factor in organ donation from brain-dead donors. Journal of transplantation. 2014;2014:296912. Epub 2014/10/29. doi: 10.1155/2014/296912 . 25349721 PMC4198775

[6] Vathsala A. Improving cadaveric organ donation rates in kidney and liver transplantation in Asia. Transplantation proceedings. 2004;36(7):1873–5. Epub 2004/11/03. doi: 10.1016/j.transproceed.2004.08.131 . 15518680

[7] Gómez MP, Irazábal MMJ, Manyalich M. INTERNATIONAL REGISTRY IN ORGAN DONATION AND TRANSPLANTATION (IRODAT)– 2019 WORLDWIDE DATA. Transplantation. 2020;104(S3):S272. doi: 10.1097/01.tp.0000699864.69759.d7

[8] (NOD-lb) NOfOaTDaT. National Organization for Organ and Tissue Donation and Transplantation (NOD-lb).

[9] (NOD-lb) NOfOaTDaT. Lebanese deceased organ procurement system.

[10] Stephan A. The Dual Aspect of Deceased Organ Donation. Experimental and clinical transplantation: official journal of the Middle East Society for Organ Transplantation. 2022;20(Suppl 1):1–2. Epub 2022/04/07. doi: 10.6002/ect.MESOT2021.L9 . 35384799

[11] Health LMoP. Hospital accreditation standards in Lebanon—Ministry of Public Health. January 2019.

[12] Zavalkoff S, Shemie SD, Grimshaw JM, Chassé M, Squires JE, Linklater S, et al. Potential organ donor identification and system accountability: expert guidance from a Canadian consensus conference. Canadian journal of anaesthesia = Journal canadien d’anesthesie. 2019;66(4):432–47. Epub 2018/12/20. doi: 10.1007/s12630-018-1252-6 . 30565159 PMC6407748

[13] Domínguez-Gil B, Delmonico FL, Shaheen FA, Matesanz R, O’Connor K, Minina M, et al. The critical pathway for deceased donation: reportable uniformity in the approach to deceased donation. Transplant international: official journal of the European Society for Organ Transplantation. 2011;24(4):373–8. Epub 2011/03/12. doi: 10.1111/j.1432-2277.2011.01243.x . 21392129

[14] Danek T, Czerwiński J, Brutkiewicz A, Kamiński A. Hospital Profiling and Hospital Stratification System as a Step for Assessment the Potential of Organ Donation From Deceased Donors. Transplantation proceedings. 2018;50(7):1975–8. Epub 2018/09/05. doi: 10.1016/j.transproceed.2018.03.124 . 30177091

[15] Potential Donor Audit Report- ODT Clinical 2022. 2022.

[16] Organ donation for transplantation: improving donor identification and consent rates for deceased organ donation. Clinical guideline [CG135]. 2016. 32091685

[17] Treatment and care towards the end of life: good practice in decision making. General Medical Council. 2022.

[18] Weiss MJ, English SW, D’Aragon F, Lauzier F, Turgeon AF, Dhanani S, et al. Survey of Canadian intensivists on physician non-referral and family override of deceased organ donation. Canadian journal of anaesthesia = Journal canadien d’anesthesie. 2020;67(3):313–23. Epub 2019/11/27. doi: 10.1007/s12630-019-01538-x . 31768789

[19] Witjes M, Jansen NE, van der Hoeven JG, Abdo WF. Interventions aimed at healthcare professionals to increase the number of organ donors: a systematic review. Critical care (London, England). 2019;23(1):227. Epub 2019/06/22. doi: 10.1186/s13054-019-2509-3 . 31221214 PMC6587298

[20] Beasley CL, Capossela CL, Brigham LE, Gunderson S, Weber P, Gortmaker SL. The impact of a comprehensive, hospital-focused intervention to increase organ donation. Journal of transplant coordination: official publication of the North American Transplant Coordinators Organization (NATCO). 1997;7(1):6–13. Epub 1997/03/01. doi: 10.7182/prtr.1.7.1.96461077u64l1m5x . 9188393

[21] de Andrade J, Figueiredo KF. Impact of Educational and Organizational Initiatives in Organ Donation in a Southern Brazilian State in the Last Decade. Transplantation proceedings. 2019;51(3):625–31. Epub 2019/04/14. doi: 10.1016/j.transproceed.2018.10.033 . 30979444

[22] Akkas M, Demir MC. Barriers to Brain Death Notifications From Emergency Departments. Transplantation proceedings. 2019;51(7):2171–5. Epub 2019/07/23. doi: 10.1016/j.transproceed.2019.02.049 . 31327476

[23] Marck CH, Jelinek GA, Neate SL, Dwyer BM, Hickey BB, Weiland TJ. Resource barriers to the facilitation of organ and tissue donation reported by Australian emergency clinicians. Australian health review: a publication of the Australian Hospital Association. 2013;37(1):60–5. Epub 2012/11/03. doi: 10.1071/AH11121 . 23116566

[24] Martínez Soba F, Masnou Burrallo N, de la Rosa Rodríguez G, Povar Marco J. [Emergency department staff and the organ donation process: recommendations from the joint working group of the National Transplant Organization and the Spanish Society of Emergency Medicine (ONT-SEMES)]. Emergencias: revista de la Sociedad Espanola de Medicina de Emergencias. 2016;28(3):193–200. Epub 2016/06/01. . 29105454

[25] Henderson SO, Chao JL, Green D, Leinen R, Mallon WK. Organ procurement in an urban level I emergency department. Annals of emergency medicine. 1998;31(4):466–70. Epub 1998/04/18. doi: 10.1016/s0196-0644(98)70255-0 9546015

[26] Garside J, Garside M, Fletcher S, Finlayson B. Utilisation of an embedded specialist nurse and collaborative care pathway increases potential organ donor referrals in the emergency department. Emergency medicine journal: EMJ. 2012;29(3):228–32. Epub 2011/03/19. doi: 10.1136/emj.2010.107334 . 21415253

[27] Ballesté C, Valero R, Istrate M, Peralta P, Mosharafa AA, Morsy AA, et al. Design and implementation of the European-Mediterranean Postgraduate Programme on Organ Donation and Transplantation (EMPODaT) for Middle East/North Africa countries. Transplant international: official journal of the European Society for Organ Transplantation. 2021;34(8):1553–65. Epub 2021/05/17. doi: 10.1111/tri.13918 . 33993570

[28] Stephan J. Public Private Partnership-based performance management in fragmented healthcare systems: the case of organ donation and transplantation in Lebanon Management de la performance basée sur un partenariat public-privé pour des systèmes de santé fragmentés: le cas du don et de la transplantation d’organes au Liban: Nantes Université; 2022.

